# Intestinal failure from thymoma-associated autoimmune enteropathy: a rare case report

**DOI:** 10.1007/s12328-025-02119-w

**Published:** 2025-04-21

**Authors:** Paris Hoey, Vishal Kaushik, Leo Francis, Niwansa Adris

**Affiliations:** 1 Department of Gastroenterology and Hepatology, Royal Brisbane and Women’s Hospital, Brisbane, QLD Australia; 2 Faulty of Medicine, University of Queensland, Brisbane, QLD Australia; 3 Pathology Queensland, Royal Brisbane and Women’s Hospital, Brisbane, QLD Australia

**Keywords:** Case report, Autoimmune enteropathy, Thymoma, Diarrhoea, Intestinal failure

## Abstract

We describe a case of altered bowel habit, early satiety, anorexia and weight loss caused by autoimmune enteropathy. This diagnosis was confirmed by biopsies obtained from upper gastrointestinal endoscopy and ileocolonoscopy. Further investigations revealed multiple pulmonary nodules which were metastases from a malignant thymoma. The patient was commenced on oral budesonide and Mesalazine, in addition to oral and enteral supplementation driven by the specialist nutrition support team, but she did not achieve histological remission, nor did she demonstrate improvement in her nutritional status. She received 3-weeks of in-patient parenteral nutrition, but then subsequently deteriorated as she declined home parenteral nutrition. The patient died eight months later from complications of her disease. This case highlights the rare manifestation of metastatic thymoma presenting with autoimmune enteropathy that led to intestinal failure and death.

## Introduction

Thymomas are rare tumours arising from thymic epithelial cells, with an incidence of 1.5 cases in a million [1]. Up to 40% of patients with thymoma will develop an autoimmune process, such as myasthenia gravis, pure red-cell aplasia and hypogammaglobulinemia [2, 3]. The pathogenesis of this phenomenon is not completely understood but likely relates to defective immune regulation from disordered thymic epithelial function. These thymic cells lack AIRE, the transcriptive factor that induces self-tolerance [4, 5].

Relapse of autoimmune manifestations of thymoma, as well as the occurrence of new processes, may emerge at any time through the entire disease continuum including after thymectomy and may herald the onset of the metastatic process. It is thought that younger age, female gender and B2 thymomas are risk factors for developing autoimmune disorders [3]. Current guidelines outline the management of thymoma-associated autoimmune disease: (a) effective tumour control with thymectomy for local, operable disease and/or chemotherapy for locally advanced and metastatic disease, (b) immunosuppressive and/or immunomodulating therapies, and (c) symptom control [2, 3].

Autoimmune processes involving the gastrointestinal tract have rarely been observed in association with thymoma. Most reported cases of thymoma-associated autoimmune enteropathy (TAIE) have acquired hypogammaglobulinemia (Good’s syndrome) or thymoma-associated multi-organ autoimmunity (TAMA) [2, 6]. TAMA presents similar to graft-versus-host disease, manifesting as a triad of chronic diarrhoea, cutaneous lesions, and abnormal liver function tests. TAIE as an entity on its own is rare, with only a few cases in the literature to date [2, 6–9]. As aforementioned, the exact pathogenesis of TAIE has not been established but it is believed to be related to disordered thymic epithelial function [4, 5]. It is unclear how tissue damage to the gastrointestinal tract occurs thereafter, however, it has been hypothesised that the increased CD8 + T lymphocytes in the lamina propria in AIE could result in direct cytotoxicity to gastrointestinal epithelial cells (10). Given some reports of successful treatment of AIE with anti-TNF-α therapy, the increased levels of TNF-α secreted from mucosal CD4 + T cells is also thought to play a role (11). Autoimmune enteropathy is also challenging to treat, with variable success with first-line therapy of corticosteroids and/or second-line immunosuppressive therapy***.*** Many patients therefore require parenteral nutrition for nutrition support [10].

Our case describes altered bowel habit, early satiety, anorexia and weight loss as a presenting complaint leading to the rare diagnosis of TAIE in the setting of metastatic thymoma. This case highlights the importance of early diagnosis, recognition of association***,*** treatment, and nutrition support in this autoimmune phenotype.

## Case report

A 51-year-old Caucasian female with a history of thymoma was referred for evaluation of altered bowel habit. Specifically, she had a 6-month history of diarrhoea occasionally alternating with constipation, as well as early satiety and anorexia. Symptoms were associated with an estimated 10 kg weight loss.

The WHO-type AB thymoma was diagnosed 4 years earlier following a presentation with autoimmune manifestations of ocular myasthenia gravis and pure red cell aplasia. This was on the background of 3 years of chronic diarrhoea, with duodenal biopsy showing partial villous atrophy with intraepithelial lymphocytosis, and random colonic biopsies with mild epithelial lymphocytosis and increased apoptosis. No gastric or terminal ileal biopsies were taken at this time, but they were reported to be macroscopically normal. She was treated with Mesalazine 4 g daily which led to partial improvement of her diarrhoea. A diagnosis of TAIE was only considered and confirmed after the discovery of the thymoma. Treatment of her thymoma included neoadjuvant chemotherapy and thymectomy with incomplete surgical margins. Her autoimmune manifestations clinically resolved after thymectomy. Therefore, her TIAE was thought to be in clinical remission however no repeat upper gastrointestinal endoscopy was performed to assess for histological remission. The patient declined further chemotherapy and then was lost to follow-up until she was referred to our gastroenterology service to investigate the recurrence of her symptoms. Her only other past medical history was depression for which she was prescribed escitalopram (a selective serotonin reuptake inhibitor (SSRI)) 10 mg daily*,* her only medication***.*** This dose of SSRI had not changed during the preceding 5 years. She was a lifelong non-smoker of cigarettes and had no history of alcohol consumption or illicit drug use. There was no family history of gastrointestinal or autoimmune conditions.

On examination, she was sarcopenic with atrophy of her temporalis, interossei and proximal limb muscles, and a body mass index (BMI) of 17.4 kg/m^2^. There was no stigmata of nutritional deficiencies. Her abdomen was non-distended and soft to palpate, without any appreciable masses. Per rectal examination did not reveal any abnormalities. The patient had a normal cardiovascular and respiratory examination. Her neurological examination, including ophthalmoscopy, was also normal with no clinical signs of fatigability to indicate the presence of myasthenia gravis.

Laboratory findings are summarised in Table 1. She had a negative faecal infective screen, but a mildly elevated calprotectin of 200 ug/g (< 50) Table 2. Serology revealed a normocytic anaemia (Hb 115 g/L) and mild cholestatic LFT derangement with a normal bilirubin (ALP 178 U/L, GGT 160 U/L, ALT 27 U/L, AST 20 U/L, bilirubin 6 umol/L). She had a low albumin of 27 g/L with borderline nutrient and micronutrient levels. Coeliac antibodies were negative, however, both intrinsic factor antibody and anti-gastric parietal cell antibody were positive. Anti-enterocyte antibodies and anti-globlet cell antibodies were unable to be obtained as this test was unavailable in the jurisdiction.

**Table 1:** Additional laboratory findings

Laboratory test	Result	Unit	Normal range
Nutrient and micronutrient screen			
Albumin	27	g/L	35–50
Potassium	3.9	mmol/L	3.5–5.2
Phosphate	1.41	Mmol/L	0.75–1.50
Magnesium	0.71	Mmol/L	0.70–1.10
Ferritin	347	ug/L	5–360
CRP	14	mg/L	< 5.0
Transferrin saturation	22	%	15–45
Retinol (Vitamin A)	1.5	umol/L	1.1–2.8
Vitamin B_12_	172	pmol/L	133–680
Vitamin C	75	umol/L	20–120
Vitamin E	26	umol/L	11–45
Folate	46.5	nmol/L	> 7.0
Zinc	7	umol/L	8–18
Thiamin diphosphate (whole blood)	0.97	nmol/g Hb	0.90–1.95
Glutathione peroxidase (red cell)	25	U/g Hb	25–62
Maganese (blood)	100	nmol/L	80–240
Ceruloplasmin/copper plasmin ratio	9.3	mol/mol	7.0–10.0
25-hydroxy-vitamin D	56	nmol/L	5–150
Immune-related screen			
Anti-nuclear antibody	Positive, 640, homogeneous	Titre	< 40
Anti-gliadin antibodies IgG	< 3	CU	< 20
Anti-tissue transglutaminase (IgA)	< 2	CU	< 20
Intrinsic factor antibody	Positive		
Anti-gastric parietal cell antibody	640	Titre	
Anti-striated muscle antibody	640	Titre	
IgA	3.4	g/L	1.0–4.0
IgG	23	g/L	7.0–16.0
IgG4	0.38	g/L	0.03–2.01
C3	0.90	g/L	0.90–1.80
C4	0.09	g/L	0.10–0.40
Total lymphocytes	2.45	10^9^/L	1.00–4.00
CD3 Absolute (%)	2.09 (85%)	10^9^/L	0.94–2.34
CD4 Absolute (%)	1.03 (42%)	10^9^/L	0.56–1.58
CD8 Absolute (%)	1.03 (42%)	10^9^/L	0.25–0.90
Infective screen			
HIV Ag/total Ab screen	Non-reactive		
Hepatitis B surface Ag (EIA)	Non-reactive		
Hepatitis C IgG screen (EIA)	Non-reactive		
CMV DNA (NAA)	Not detected		
EBV IgG and IgM (EIA)	Reactive		
Toxo gondii IgG (EIA)	Non-reactive

**Table 2:** Additional faecal tests

Faecal tests	Result	Unit	Normal range
Bacterial PCR	Negative		
Protozoan PCR	Negative		
*Clostridioides difficile* toxin gene PCR	Negative		
Calprotectin (faeces)	200	ug/g	< 50
Pancreatic elastase	220, soft	ug/g faeces	> 200

**Fig. 1:**
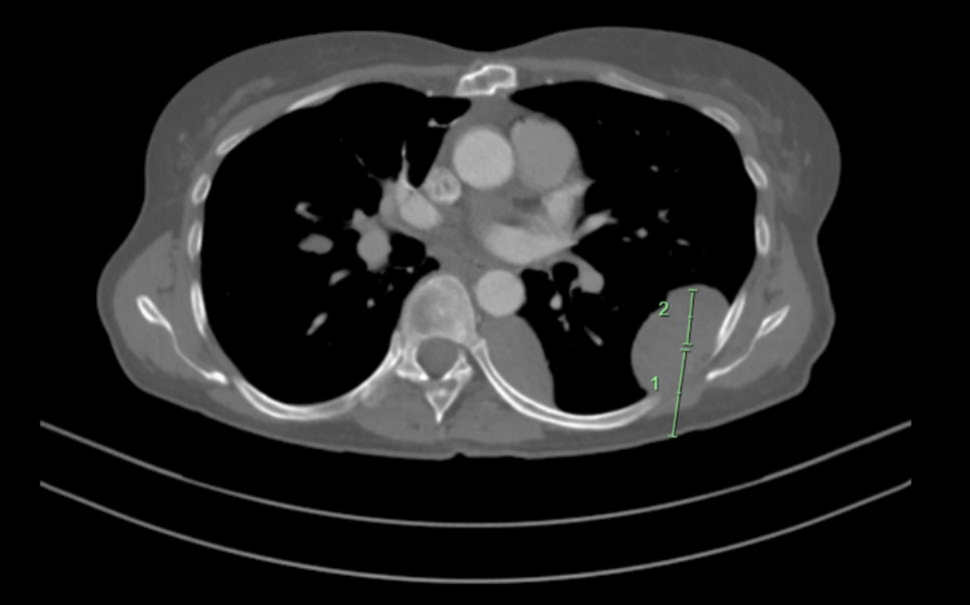
CT-chest with contrast demonstrating the largest subpleural mass in the left lower lobe superior segment measuring 36 mm by 22 mm

**Fig. 2:**
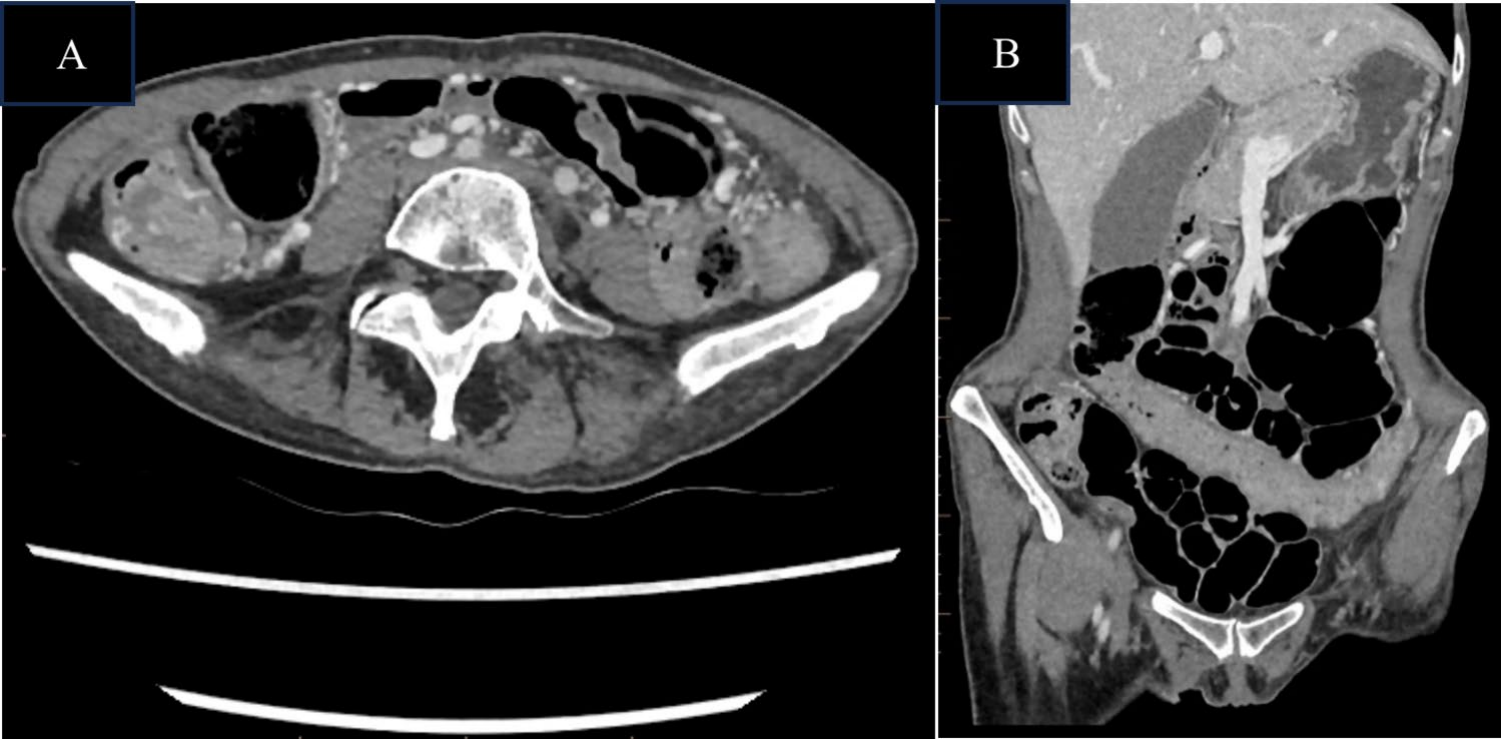
CT-abdomen and pelvis with intravenous contrast shows no gross abnormalities of the gastrointestinal tract within the limits of the study. **A** Axial plane. **B** Coronal plane

Given the history of thymoma with incomplete resection margins, a computed tomography (CT) scan of the chest, abdomen and pelvis was obtained to look for disease recurrence as the potential cause for weight loss. The CT demonstrated the presence of numerous bilateral subpleural lobulated masses (Fig. 1), but no gross abnormalities of the gastrointestinal tract (Fig. 2). A CT-guided biopsy of the pleural mass confirmed the diagnosis of metastatic thymoma.

**Fig. 3:**
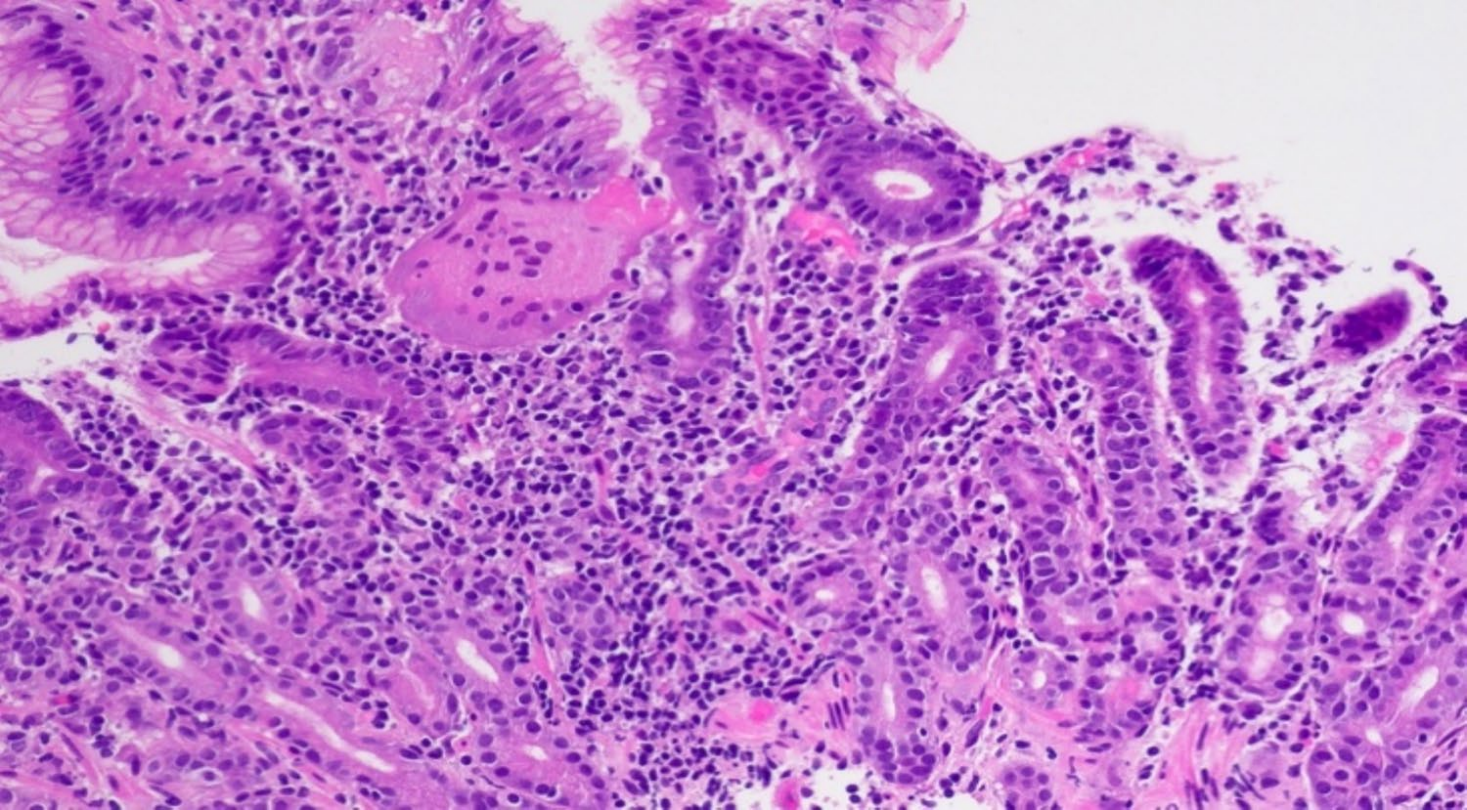
Gastric corpus mucosa with dense lymphocyte infiltrate with a loss of parietal cells (hematoxylin and eosin × 200)

**Fig. 4:**
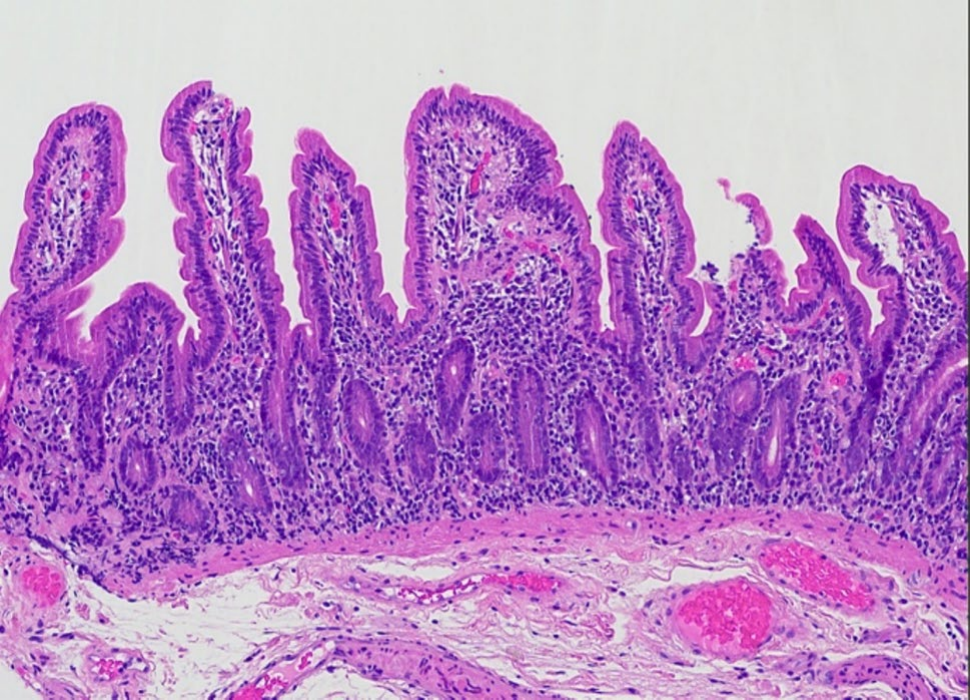
Duodenal biopsy showing absence of goblet cells and paneth cells, with partial villous blunting (hematoxylin and eosin × 100)

**Fig. 5:**
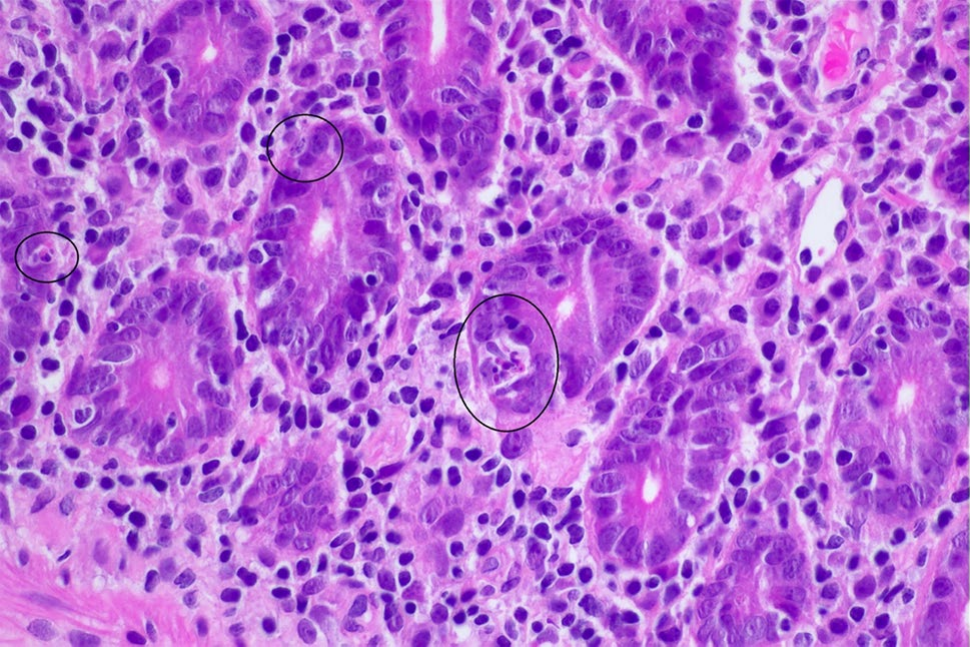
High power view (hematoxylin and eosin × 200) of the duodenal crypt epithelium, highlighting several apoptotic bodies (black circles)

**Fig. 6:**
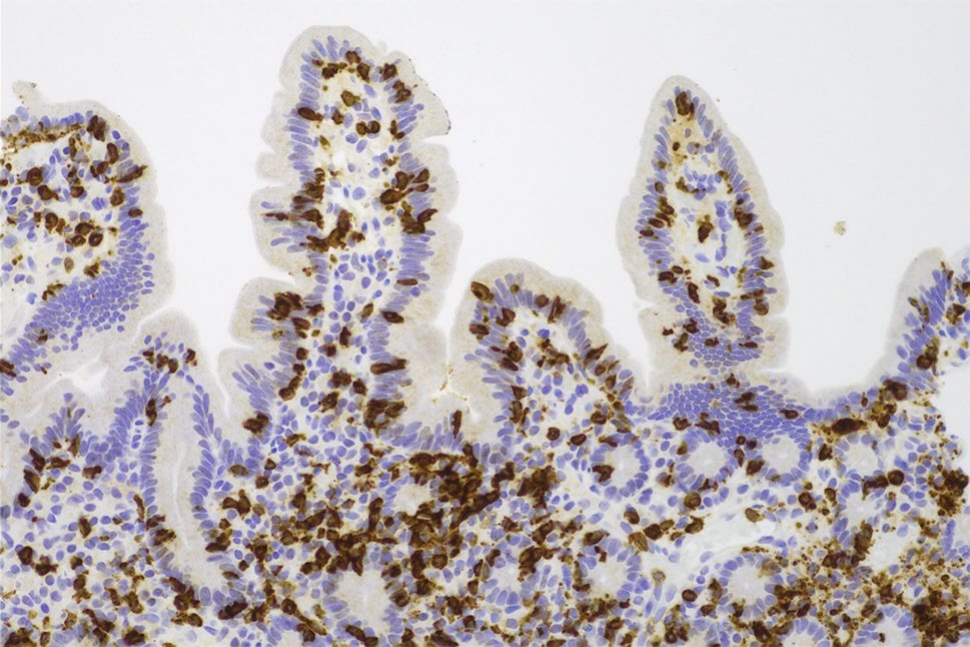
CD8 immunohistochemistry demonstrating increased intraepithelial CD8-positive T-lymphocytes in the duodenal crypt and surface epithelium (× 200)

An upper gastrointestinal endoscopy and ileocolonoscopy were performed and reported as showing mild erythema of the gastric corpus and mild villous atrophy of the duodenum, with a normal appearing terminal ileum, and colon. This bidirectional endoscopy, as well as the index endoscopy, were performed at other facilities and therefore endoscopic images are unavailable. Biopsies from the gastric corpus (Fig. 3) showed a dense infiltrate of lymphocytes, plasma cells with some Russell bodies, and focal neutrophils. Intraepithelial CD8-positive lymphocytes were also present. There was an absence of parietal cells. A small number of multinucleated giant cells were present within the lamina propria and in the lumen of occasional gastric pits. *Helicobacter* species were not identified. The gastric antral mucosa showed similar inflammatory features. The duodenal biopsy (Fig. 4) showed partial villous atrophy with increased lymphocytes and plasma cells in the lamina propria. The crypt epithelium contained increased intraepithelial CD8-positive T-lymphocytes and a small number of apoptotic bodies (Figs. 5 and 6). There was an absence of both goblet cells and Paneth cells. *Giardia* species were not seen. Similar features were seen in the terminal ileum but differed by a severe degree of villous blunting, conspicuous crypt apoptosis and a prominent reactive lymphoid follicle. Random biopsies from the right and left colon showed normal mucosal architecture with increased plasma cells and intraepithelial lymphocytes, conspicuous apoptosis, and a complete absence of goblet cells.

Based on the patient’s history and histopathological findings of the stomach, duodenum, terminal ileum and colon, we considered her disease condition to be that of recurrent TAIE.

The patient was commenced on budesonide 9 mg daily and oral Mesalazine 1 g twice daily for the treatment of AIE, with the latter initiated because the patient found that the medication improved the severity of her diarrhoea at the time her index diagnosis was made pre-operatively. Micronutrient levels were optimised with oral zinc, thiamine and multivitamin supplementation.

**Fig. 7:**
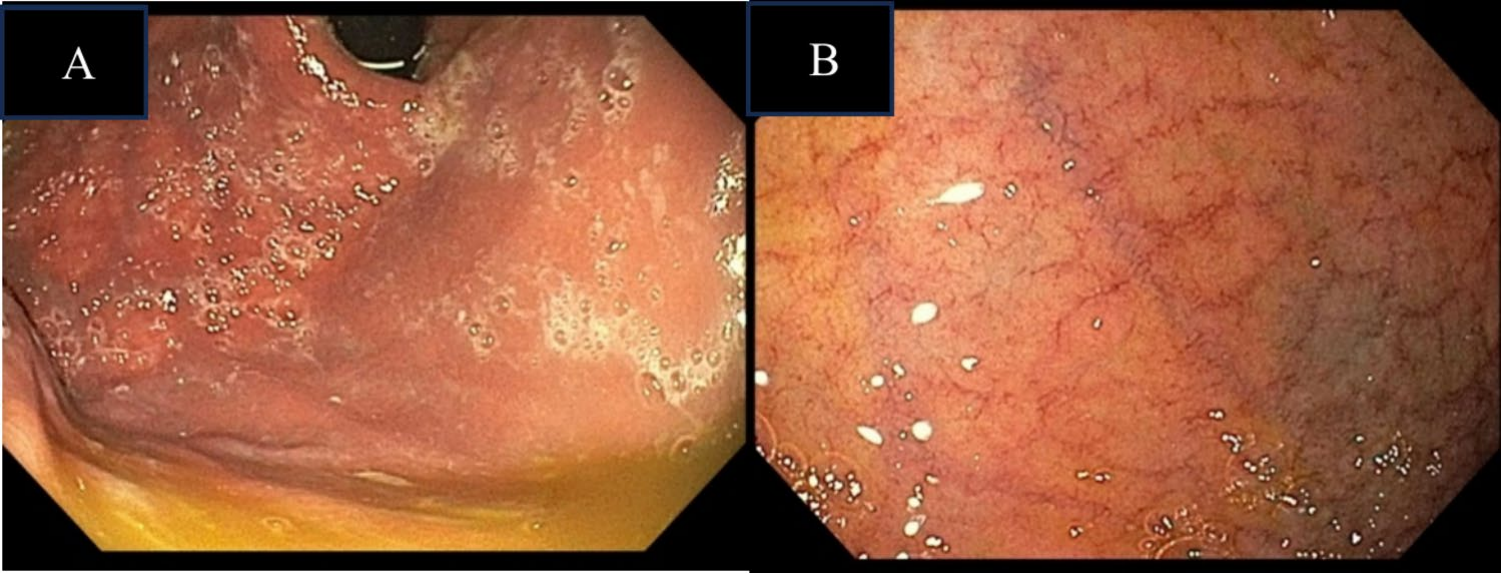
Endoscopic images following 4 months of budesonide therapy for TAIE. **A** Image of the gastric body showing moderate gastritis. **B** Image of the second part of the duodenum (D2) showing mucosal flattening

At four months, her diarrhoea, early satiety and anorexia gradually became worse. Her nutrition continued to deteriorate thus resulting in worsening sarcopenia, reduced hand-grip strength and cognitive impairment with short-term memory loss. Biochemically, her serum albumin failed to correct, and her BMI dropped further to 13.4 kg/m^2^. A repeat upper gastrointestinal endoscopy was performed to assess disease activity and showed progression of disease macroscopically with moderate gastritis (Fig. 7A) and villous atrophy of the duodenum (Fig. 7B). Gastric and duodenal biopsies demonstrated findings consistent with the previous biopsies, in keeping with TAIE, but of increased severity. The gastric biopsy showed moderate chronic inflammation with a diffuse lymphoplasmacytic infiltrate of the lamina propria. Dudoenal biopsy showed moderate villous blunting with increased intraepithelial lymphocytes and prominent plasma cells, in the absence of goblet cells and Paneth cells. Therefore, she was determined to have refractory TAIE to her current treatment.

Supplemental high-protein and high-energy oral and nasogastric tube nutrition were prescribed based on her calculated requirements. She failed to demonstrate improvement in her weight and nutritional parameters despite 4 weeks of good adherence to oral and enteral nutrition. She was therefore diagnosed with intestinal failure and admitted to the hospital for parenteral nutrition for nutritional stabilisation. A 4 kg weight gain was noted after 3 weeks of parenteral nutrition initiation in combination with ongoing oral/enteral nutrition. Long-term parenteral nutrition was declined by the patient due to concerns about the potential complications and limitations on her lifestyle. She asked to be discharged from the hospital.

Due to her sarcopenia and poor Eastern Cooperative Oncology Group (ECOG) performance score of 2, the patient was not considered safe for chemotherapy for her metastatic thymoma. She continued to deteriorate and died a few months later due to multi-organ failure during an admission with refractory *Clostridiodes difficile* colitis.

## Discussion

We present a rare case of metastatic thymoma that manifested with recurrence of gastrointestinal symptoms and severe malnutrition from intestinal failure attributable to thymoma-associated autoimmune disease. Our case highlights the heterogenous nature of thymoma manifestations, as the patient initially presented with myasthenia gravis, pure red cell aplasia and TAIE and had a recurrence of TAIE subsequently as a manifestation of metastatic disease. To the best of our knowledge, this is the first reported case of TAIE relapse due to thymoma recurrence. The diagnosis and treatment challenges of TAIE are discussed herein.

AIE as a cause of intractable diarrhoea is an important but rarely encountered differential diagnosis. Our patient’s history of previous thymoma and previous TAIE raised the clinical suspicion of relapsed disease, which was later confirmed by histopathological findings. She met the proposed diagnostic criteria for AIE: (a) chronic diarrhoea, (b) malabsorption, (c) specific small bowel histology of villous atrophy with the absence of goblet and Paneth cells, deep crypt lymphocytosis, increased crypt apoptotic bodies and minimal intra-epithelial lymphocytosis (Fig. 4), and (d) exclusion of other causes of villous atrophy. These histopathological changes typically affect the entire intestinal tract in AIE, which was confirmed by biopsies of the stomach and colon in our patient [11]. The widespread distribution of histologic findings excludes a differential diagnosis of coeliac disease, which is limited to the small bowel. Anti-enterocyte or anti-goblet cell antibodies were not tested in our case, but their absence does not exclude the diagnosis of AIE. Our patient had no dermatologic findings, as well as preserved immunoglobulin levels, arguing against a diagnosis of Good’s syndrome or TAMA. In addition, to our knowledge, there have been no descriptions of SSRI-related enterocolitis in the literature. SSRIs are a recognised cause of microscopic colitis, and while the colonic biopsies did contain increased intraepithelial lymphocytes that can be seen in microscopic colitis, the presence of conspicuous apoptotic bodies, complete absence of goblet cells and similar findings in the stomach and small bowel, are not features of that diagnosis. The initial clinical improvement in symptoms at index diagnosis despite continued SSRI intake, as well as the fact that there was no change in her dose when her symptoms recurred, again goes against a medication-related cause of enterocolitis. Finally, even in the presence of clinically significant diarrhoea and malnutrition associated with AIE, cross-sectional imaging is often unremarkable, as occurred in our case, with only up to 40% of cases having prominent mesenteric lymph nodes as their only radiological finding [12]. It is also important to note that the paucity of intra-abdominal fat, as well as the lack of luminal distension due to the absence of oral contrast in our patient’s CT modality, would have limited the assessment of the gastrointestinal tract.

There are no established guidelines for the treatment of TAIE, however, there are several case studies that report success in AIE with the use of corticosteroids (either prednisone or budesonide) [11, 13]. Unfortunately, up to two-thirds of these patients who achieve remission will become steroid-dependent or refractory [11]. Budesonide was chosen in our case given its high first-pass metabolism and reduced side effect profile. Mesalazine, which acts locally on colonic mucosa, was used as an adjunct. Although 5-aminosalicylate drugs have not been described as treatment for this condition, it was thought to be worth trialling as it is a low-risk drug with proven benefits in other autoimmune gastrointestinal diseases (namely ulcerative colitis). Unfortunately, the combined use of budesonide and Mesalazine in our patient did not induce histological remission by four months. Other agents including azathioprine, 6-mercaptopurine, cyclosporin, tacrolimus, mycophenolate mofetil, sirolimus, infliximab, rituximab and vedolizumab have shown variable success rates in the treatment of AIE [11, 14–19]. Given our patient’s history of metastatic thymoma and severe malnutrition that would render her at high risk of infection, as well as the unknown effects of strong immunosuppression on tumour progression, we were not inclined to pursue this therapy.

Intestinal failure is defined as “the reduction of gut function below the minimum necessary for the absorption of macronutrients and/or water and electrolytes, such that intravenous supplementation is required to maintain health and/or growth” [20]. Home parenteral nutrition, the treatment of choice for patients with intestinal failure, was recommended for our patient due to persistent malnutrition despite an adequate trial of oral and enteral nutrition. The patient’s informed decision to reject the utilisation of home parenteral nutrition to treat malnutrition was a major contributor to her clinical deterioration and subsequent death. Therefore, early recognition and reversal of malnutrition is a vital aspect of disease treatment.

Our patient had biopsy-proven autoimmune pangastritis, in the setting of anti-gastric parietal cell and intrinsic factor antibodies. This differs from the few cases of thymoma-associated autoimmune gastritis in the literature that have all occurred in the absence of autoantibodies [21, 22]. There are a few key factors in our case which clearly indicate that it is not an uncomplicated conventional autoimmune gastritis (AIG) despite having AIG autoantibodies: (1) involvement not limited to the corpus despite anti-gastric parietal cell antibodies, and (2) an absence of neuroendocrine cell hyperplasia and intestinal metaplasia. Therefore, it is difficult to ascertain whether the gastric involvement represents dual pathology of usual AIG and AIE, or part of the thymoma-related process.

The long-term outcome of patients with gastrointestinal autoimmune manifestations in the setting of metastatic thymoma is scarce. While surgical and/or chemotherapy options exist for metastatic thymoma, with a relatively good prognosis (38% 5-year relative survival rate), the safety and durability of treatment options may be limited by the emergence and complications of autoimmune diseases, as occurred in our case [23].

In summary, we describe a patient with severe gastrointestinal symptoms leading to intestinal failure from AIE, in the setting of a previous thymoma with incomplete margins, that led to the diagnosis of metastatic disease. Although autoimmune manifestations may emerge at any time through the disease continuum, relapse of symptoms requires the exclusion of tumour recurrence. TAIE is an important but rarely encountered phenomenon of thymoma, and this case reinforces the importance of a high index of suspicion, early endoscopic assessment, treatment, and nutritional support.

## Abbreviations

**TAIE**: Thymoma-associated autoimmune enteropathy
**TAMA**: Thymoma-associated multi-organ autoimmunity
**BMI**: Body mass index
**CT**: Computed tomography
**AIE**: Autoimmune enteropathy
**ANA**: Anti-nuclear antibody
**ANCA**: Anti-neutrophil cytoplasmic antibodies
**ENA**: Extractable nuclear antigen antibodies
**ECOG**: Eastern Cooperative Oncology Group
**AIG**: Autoimmune gastritis

## References

[1] Tayabali K, Pothiwalla H, Thole J, et al. Rarity among the rare-large and invasive thymoma, a case report and review. J Community Hosp Intern Med Perspect. 2020;10:233–7. 32850071 10.1080/20009666.2020.1766819 PMC7426983

[2] Riquelme P, Tisch A, Liang Y, et al. Thymoma-associated chronic diarrhea: a case of autoimmune enteropathy. Cancer Treat Res Commun. 2015;4:46–9.

[3] Blum T, Misch D, Kollmeier J, et al. Autoimmune disorders and paraneoplastic syndromes in thymoma. J Thorac Dis. 2020;12:7571–90. 33447448 10.21037/jtd-2019-thym-10 PMC7797875

[4] Mais D, Mulhall B, Adolphson K, et al. Thymoma-associated autoimmune enteropathy. Am J Clinc Pathol. 1999;112:810–5. 10.1093/ajcp/112.6.810 10587704

[5] Fidas P, Long A, Fintelmann F, et al. Case 31–2015: A 29-year-old man with thymoma, diarrhea, and weight loss. N Engl J Med. 2015;373(15):1458–67. 26444733 10.1056/NEJMcpc1406663

[6] Ishiguro F, Kawaguchi K, Mizuno T, et al. Invasive thymoma with autoimmune gastroenteropathy. Int Canc Conf J. 2014;3:104–7.

[7] Alaber O, Elturki S, Buaisha H. Autoimmune enteropathy (AIE) in a patient diagnosed with thymoma: a case report. Am J Gastroenterol. 2022;117:1642.

[8] Robbins G, Tracht J, Davis D, et al. New treatment option for autoimmune enteropathy: a rare case of intractable diarrhea treated with vedolizumab. ACG Case Rep J. 2018;5:92. 10.14309/crj.2018.92 PMC6358579 30775395

[9] Gu B, Liu K, McKenzie C, et al. Gastrointestinal: autoimmune enteropathy associated with thymoma. J Gastroenterol Hepatol. 2022;37:606. 34632628 10.1111/jgh.15692

[10] Ciccocioppo R, D’Alo S, Sabatino A, et al. Mechanisms of villous atrophy in autoimmune enteropathy and coeliac disease. Clin Exp Immunol. 2002;128:88–93. 11982595 10.1046/j.1365-2249.2002.01795.x PMC1906355

[11] McCarthy D, Katz S, Gazze L, et al. Selective IgA deficiency associated with total villous atrophy of the small intestine and an organ-specific anti-epithelial cell antibody. J Immunol. 1978;120:932–8. 632592

[12] Paul J, Shihaz A. Autoimmune enteropathy in adults. Adv Dig Med. 2020;9:75–81.

[13] Gentile N, Murray J, Pardi D. Autoimmune enteropathy: a review and update of clinical management. Curr Gastroenterol Resp. 2012;14:380–5. 10.1007/s11894-012-0276-2 PMC3912565 22810979

[14] Akram S, Murray J, Pardi D, et al. Adult autoimmune enteropathy: Mayo Clinic Rochester experience. Clin Gastroenterol Hepatol. 2007;5:1282–90. 17683994 10.1016/j.cgh.2007.05.013 PMC2128725

[15] Elwing J, Clouse R. Adult-onset autoimmune enteropathy in the setting of thymoma successfully treated with infliximab. Dig Dis Sci. 2005;50:928–32. 15906770 10.1007/s10620-005-2666-x

[16] Oliva-Hemker M, Loeb D, Abraham S, et al. Remission of severe autoimmune enteropathy after treatment with high-dose cyclophosphamide. J Pediatr Gastroenterol Nutr. 2003;36:639–43. 12717089 10.1097/00005176-200305000-00010

[17] Von Hahn T, Stopik D, Koch M, et al. Management of severe refractory adult autoimmune enteropathy with infliximab and tacrolimus. Digestion. 2005;71:141–4. 15785040 10.1159/000084648

[18] Sanderson R, Phillips A, Spencer J, et al. Response to autoimmune enteropathy to cyclosporin A therapy. Gut. 1991;32:1421–5. 1752480 10.1136/gut.32.11.1421 PMC1379182

[19] Gupta A, Yilmaz O, Fisher M, et al. Abatacept: a new treatment option for refractory adult autoimmune enteropathy. J Clin Gastroenterol. 2014;48:55–8. 24045285 10.1097/MCG.0b013e3182a4e0ec PMC4518556

[20] Pironi L. Definitions of intestinal failure and the short bowel syndrome. Best Pract Res Clin Gastroenterol. 2016;30:173–85. 27086884 10.1016/j.bpg.2016.02.011

[21] Hashizume T. Good’s syndrome and Pernicious anemia. Curr Gastroenterol Resp. 2012;14:380–5.

[22] Robins-Browne R, Green R, Katz J, et al. Thymoma, pure red cell aplasia, pernicious anaemia and candidiasis: a defect in immunohomeostasis. Br J Haematol. 1977;36:5–13. 871425 10.1111/j.1365-2141.1977.tb05749.x

[23] Cancer Net [Internet]. American Society of Clinical Oncology. Thymoma and thymic carcinoma: Statistics; 2023 March [cited 2024 March 21]; Available from: https://www.cancer.net/cancer-types/thymoma-and-thymic-carcinoma/statistics#:~:text=If%20the%20cancer%20has%20spread%20to%20distant%20parts%20of%20the,carcinoma%20cancer%20every%205%20years.

